# Systematic review of invasive meningococcal disease epidemiology in the Eastern Mediterranean and North Africa region

**DOI:** 10.1186/s12879-021-06781-6

**Published:** 2021-10-22

**Authors:** Alp Giray Dogu, Anouk M. Oordt-Speets, Femke van Kessel-de Bruijn, Mehmet Ceyhan, Amine Amiche

**Affiliations:** 1 Sanofi Pasteur, Dubai, UAE; 2 Pallas, Rotterdam, the Netherlands; 3 Faculty of Medicine, Hacettepe University, Ankara, Turkey

**Keywords:** Invasive meningococcal disease, Epidemiology, Disease burden, EMNA region, Eastern Mediterranean, North Africa, Systematic review

## Abstract

**Background:**

Invasive meningococcal disease (IMD) represents a global health burden. However, its epidemiology in the Eastern Mediterranean (EM) and North Africa (NA) regions is currently not well understood. This review had four key objectives: to describe asymptomatic meningococcal carriage, IMD epidemiology (e.g. serogroup prevalence, case-fatality rates [CFRs]), IMD presentation and management (e.g. clinical diagnosis, antibiotic treatments) and economic impact and evaluation (including health technology assessment [HTA] recommendations) in EM and NA.

**Methods:**

A systematic literature search (MEDLINE and EMBASE) was conducted (January 2000 to February 2021). Search strings included meningococcal disease and the regions/countries of interest. Identified publications were screened sequentially by title/abstract, followed by screening of the full-text article; articles were also assessed on methodological quality. Literature reviews, genetic sequencing or diagnostic accuracy studies, or other non-pertinent publication type were excluded. An additional grey literature search (non-peer-reviewed sources; start date January 2000) was conducted to the end of April 2019.

**Results:**

Of the 1745 publications identified, 79 were eligible for the final analysis (n = 61 for EM and n = 19 for NA; one study was relevant to both). Asymptomatic meningococcal carriage rates were 0–33% in risk groups (e.g. military personnel, pilgrims) in EM (no data in NA). In terms of epidemiology, serogroups A, B and W were most prevalent in EM compared with serogroups B and C in NA. IMD incidence was 0–20.5/100,000 in EM and 0.1–3.75/100,000 in NA (reported by 7/15 countries in EM and 3/5 countries in NA). CFRs were heterogenous across the EM, ranging from 0 to 57.9%, but were generally lower than 50%. Limited NA data showed a CFR of 0–50%. Data were also limited in terms of IMD presentation and management, particularly relating to clinical diagnosis/antibiotic treatment. No economic evaluation or HTA studies were found.

**Conclusions:**

High-risk groups remain a significant reservoir of asymptomatic meningococcal carriage. It is probable that inadequacies in national surveillance systems have contributed to the gaps identified. There is consequently a pressing need to improve national surveillance systems in order to estimate the true burden of IMD and guide appropriate prevention and control programmes in these regions.

**Supplementary Information:**

The online version contains supplementary material available at 10.1186/s12879-021-06781-6.

## Background

*Neisseria meningitidis,* a bacterium found exclusively in humans, poses a global health risk [1]. Infection with *N. meningitidis* usually results in asymptomatic carriage, in which the bacteria colonise the naso-oropharyngeal mucosa [2–4]. Globally, carriage prevalence varies both by region and age group [2, 5]. International estimates indicate that carriage prevalence ranges from 4.5% in infants to 7.7% in children aged 10 years, peaking at 23.7% in adolescents (19 years) and decreasing to 7.8% by the age of 50 years [2, 5]. It should be noted, however, that individual regions may have different carriage age-distributions [5, 6]. Although the majority of *N. meningitidis* carriages are cleared spontaneously (i.e. without treatment), in some cases the pathogen may pass through the naso-oropharyngeal mucosa, entering the bloodstream. Persistence of *N. meningitidis* in the bloodstream may subsequently result in invasive meningococcal disease (IMD) [2, 4].

IMD is the most severe type of meningococcal infection, with common clinical manifestations including meningitis and meningococcaemia [1]. Approximately 1.2 million IMD cases occur annually worldwide, and it is associated with a case-fatality rate (CFR) of approximately 10% [1, 7]. However, the CFR may increase significantly in cases of inadequate or delayed diagnosis and treatment, and varies according to serogroup [8, 9]. Atypical clinical presentation has been observed for various serogroups (e.g. acute gastrointestinal symptoms for serogroup W, primary pneumonia for serogroup Y, septic arthritis for serogroups C and W); such cases may be misdiagnosed, potentially leading to a high CFR [1, 8–10]. The incidence of IMD, which may occur as sporadic, endemic or epidemic infection, is influenced by various factors. These include microbial factors (e.g. virulence), host susceptibility factors (e.g. age, medical conditions) and environmental factors such as geographical location (including travel to endemic/epidemic areas), seasonal variations, and mass gatherings [1, 7, 11–14]. Globally, the incidence of IMD is highest in infants and young children, with over 75% of all cases of meningococcal meningitis and meningococcaemia occurring in children aged < 5 years. However, a second, smaller peak of incidence has been observed in adolescents and young adults [1, 15]. In addition, the CFR is age dependant, and is highest in older adults (aged ≥ 65 years) [16, 17].

The majority of cases of IMD are caused by serogroups A, B, C, W and Y [1, 7, 11]. Given the unpredictability of IMD, proactive vaccination strategies are considered the best method to ensure population-wide protection [18]. Currently available vaccines provide coverage for serogroups A, B, C, W and Y; quadrivalent vaccines cover the serogroups A, C, W and Y, while mono-/bivalent vaccines are available for prevention of disease caused by serogroups A, B and C [18]. Two pentavalent vaccines (ABCWY and ACYWX) are also currently in development [19, 20].

IMD is often associated with situations in which a high degree of crowding occurs, which includes events such as the Umrah and Hajj, which are mass gatherings of Muslim pilgrims in Saudi Arabia [11, 14, 21]. Attendance at the Hajj in particular may exceed 1 million non-resident attendees [22]. These gatherings have previously been associated with local and international outbreaks of IMD, as many pilgrims who attend Umrah/Hajj travel to Saudi Arabia from the African meningitis belt [13]. The latter is a geographical region stretching from Senegal to Ethiopia that has the highest burden of IMD in the world [6, 23]. It is thought that this mass movement of pilgrims may influence the epidemiology of IMD in the Eastern Mediterranean (EM) and North Africa (NA) regions (referred to collectively as the EMNA region), allowing different serogroups to spread between regions and potentially resulting in local outbreaks of disease [24]. Despite these concerns, however, data on IMD are sparse or lacking in the EMNA region as a whole. Various surveillance systems (ranging from developed to suboptimal) are present in the African meningitis belt, Algeria, Morocco and Turkey, but few other countries in the region have established this type of infrastructure [25].

The aim of this systematic review is to describe asymptomatic meningococcal carriage, IMD epidemiology, IMD presentation and management, and economic impact and evaluation in the EMNA region.

## Methods

### Objectives

The key objectives of this study were to review the following in the EMNA region: (1) asymptomatic meningococcal carriage; (2) IMD epidemiology (serogroup distribution, incidence, CFRs and complications and sequelae); (3) IMD presentation and management (clinical presentation, hospitalisation, antibiotic treatment and prophylaxis/vaccination); (4) and economic impact and evaluation (including health technology assessment [HTA] recommendations).

### Systematic literature search

A systematic review of the literature was performed following the Cochrane Collaboration and Preferred Reporting Items for Systematic Reviews and Meta-Analyses (PRISMA) guidelines [26]. A literature search of the MEDLINE and EMBASE databases was completed with date limits of 1 January 2000 to February 2021. The search strings included English-language terms for meningococcal disease, and the regions and countries of interest. Full details of the PRISMA checklist and the systematic review search strategy (including formulation of review questions, literature searches, selection procedure, data extraction and quality control) are included in Additional file 1. Unless otherwise specified, dates and date ranges stated in the Results section indicate the time period during which the data contained in individual publications were collected, and not the year of publication.

The original search was conducted in the Asia, Middle East and Eurasia (AMEE) region. Given the importance of meningitis in the EMNA region, we have chosen to focus solely on studies relevant to that region in this manuscript. As mass gatherings of Muslim pilgrims are a regular occurrence in the EMNA region, Eurasian countries proximal to the Middle East with a majority Muslim population (i.e. Pakistan, Turkey) were also included in the EM region. For the purposes of this review, the EMNA region was divided into two sections (EM and NA). The EM region was defined as Bahrain, Iran, Iraq, Jordan, Kuwait, Lebanon, Oman, Pakistan, Palestinian Territories, Qatar, Saudi Arabia, Syria, Turkey, United Arab Emirates, and Yemen. The NA region was defined as Algeria, Egypt, Libya, Morocco, and Tunisia.

### Study screening

Details of the search strategy can be found in Additional file 1. Selection was not limited by language of the publication. Publications identified by the search were screened sequentially by title and abstract, followed by screening of the full-text article. Literature reviews, genetic sequencing or diagnostic accuracy studies, or other non-pertinent publication type were excluded. As most studies were not of a classical design suited to appraisal using existing standardised checklists, such as surveillance studies or cross-sectional studies, no checklists were used to assess the quality of the articles or to calculate a total quality score. Nevertheless, articles were assessed on their methodological quality without standardised checklists and one article was excluded because of major limitations in their design. To fill the gaps from the peer-reviewed literature, a grey literature search (i.e. hand search) was conducted in April 2019. Key websites (listed in Additional file 1) were searched using English search terms for relevant grey literature documents, conference abstracts and other data sources (including other websites) dating from January 2000 to April 2019.

### Data extraction

Data on the key objectives were extracted and stratified by age group (children only [defined per study; variable age range], adults only, and children and adults), serogroup and population (general population, military, students, pilgrims, and household contacts of individuals with IMD) using a standard Excel spreadsheet. No formal assessment of publication bias was performed.

## Results

**Fig. 1:**
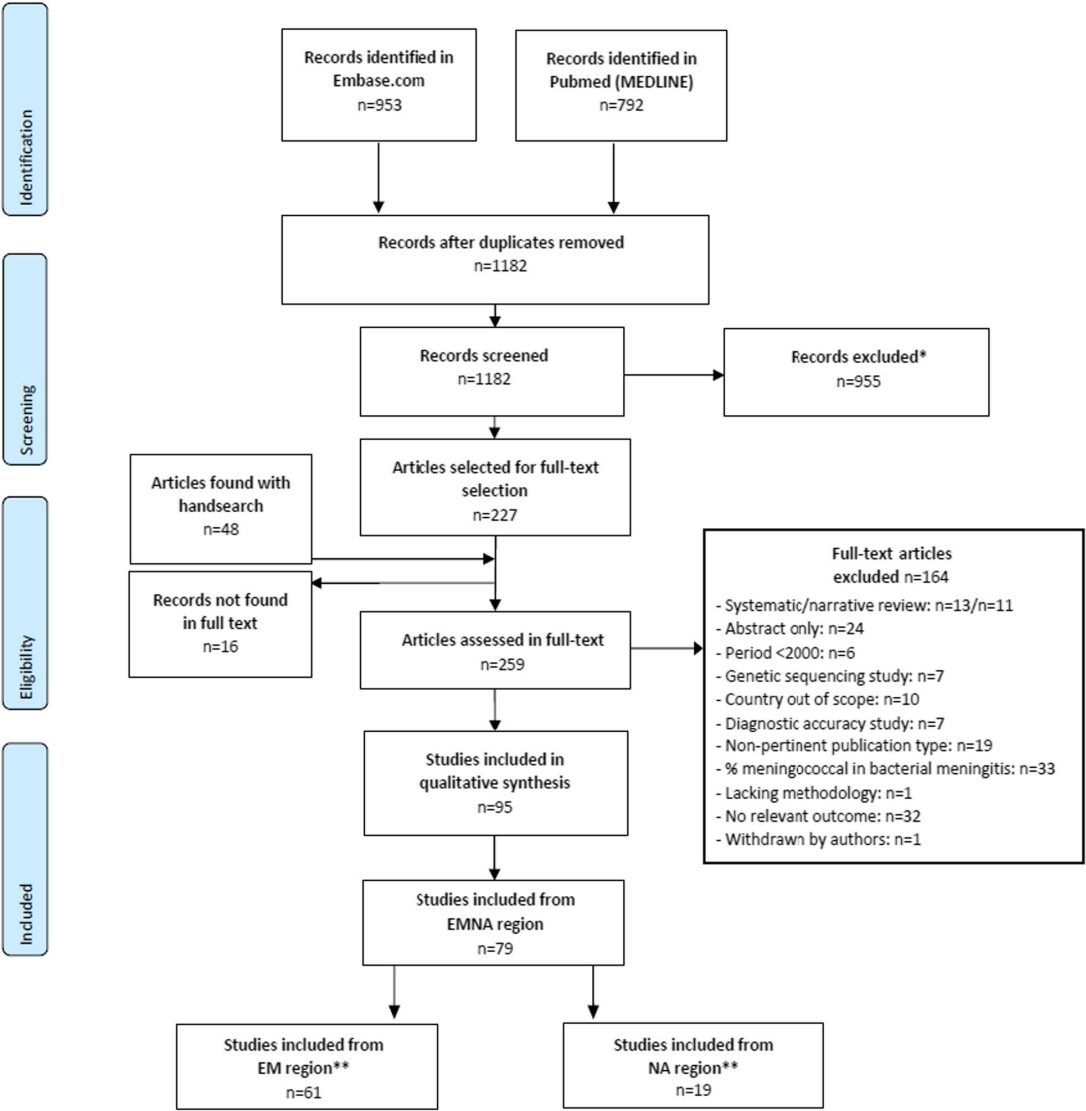
Systematic review flow diagram (including number of records and final studies selected for inclusion). *Records were excluded due to a lack of data relevant to the objectives of the current study. **One study spanned both the EM and NA regions and so was counted in both.

The literature search identified a total of 1745 publications, of which 563 were duplicates (Fig. 1).

A total of 955 publications were excluded as they did not include data relevant to the objectives of this study, leaving 227 articles for full-text screening. An additional 48 articles were identified by hand search, and 16 articles were excluded as they were not found in full text. Following full-text screening of the remaining 259 articles, 164 articles were excluded as they did not meet the inclusion criteria (further details can be found in Fig. 1). A total of 95 articles were then considered for inclusion (i.e. included in qualitative synthesis) in the systematic literature review. Of these, 79 were relevant to the EM and NA regions; (n = 61 and n = 19 respectively, with one study spanning both regions).

Data were unavailable for objective 4 (economic impact and evaluation). Data for objectives 1–3 (asymptomatic meningococcal carriage, IMD epidemiology and IMD presentation and management, respectively) were not always available for all countries, and overall, there were fewer relevant publications identified in the NA versus the EM region (Additional file 1: Fig. S1).

## Objective 1: asymptomatic meningococcal carriage

Here, we report the asymptomatic meningococcal carriage by age group (children, adults, and adults or children) in the general population or by risk group (household contact, military, pilgrim or student) in the EMNA region. Data were only available in the EM region (Table 1).

**Table 1:** Asymptomatic carriage rate of meningococcal disease (EM region only)

References	Study design	Setting (region)	Population Total number of swabbed persons	Asymptomatic carriage rate (%)		
				Year	Age category (age range)	Serogroup
Turkey [27]	Cross-sectional study	6 well child clinics, 11 day-care centres and 7 elementary schools (Anatolian side of Istanbul)	General population N = 1382	2000: 1.2	Children (0–10 y): 1.2	All: 1.2 - A: 0.1 - B: 0.4 - C: 0.0 - W135: 0.1 - Y: 0.7 - Other: 0.1
Turkey [28]	Cross-sectional study	Primary schools in the city centre (Manisa)	General population N = 1128	2001–2002: 4.8–8.0	Children (7–14 y): 4.8–8.0	All: 4.8–8.0 - A: 1.8 - B: 1.4 - C: 2.2 - W135: 0.7 - Other: 0.2
Turkey [29]	Cross-sectional study	Marmara University Pendik Training and Research Hospital (Istanbul)	General population N = 1000	2012–2013: 0.6	Children and adults (0–79 y): 0.6	All: 0.6 - B: 0.5 - Other: 0.1
Turkey [31]	Cross-sectional study	13 sites (12 Turkish provinces)	General population N = 1518	2015: 2.8–11.0	Children (10–17 y): 2.8–11.0	All: 2.8–11.0 - A: 0.3 - B: 0.6 - C: 0.0 - W135: 4.2 - Y: 0.3 - Other: 0.9
					Adults (18–20 y): 3.1–10.3	
					Children and adults (10–24 y): 6.3	
Turkey [32]	Cross-sectional study	Ýnönü University Faculty of Medicine hospital (Malatya)	General population N = 77	2006: 1.3	Adults (18–62 y): 1.3	All: 1.3
Turkey [30]	Cross-sectional study	City (Diyarbakir)	General population N = 255	2004: 2.4–2.8	Children (0–14 y): 2.8	All: 2.4–2.8 - W135: 0.4 - Other: 0.4
					Adults (20–70 y): 2.5	
Iran [33]	Longitudinal study	Shiraz airport before and after the Hajj (Shiraz)	Pilgrims N = 674 (before Hajj 2003) N = 674 (post Hajj 2003) Hajj study	Before Hajj 2003: 5.2	Adults (NR): 5.2	All: 5.2 - A: 0 - B: 1.3 - C: 0.7 - W135: 0.1 - Y: 0.7 - Other: 0.0–1.5
				Post Hajj 2003: 4.6	Adults (NR): 4.6	All: 4.6 - A: 0.1 - B: 0.3 - C: 0.1 - W135: 0.1 - Y: 0.3 - Other: 0.0–3.1
Iran [34]	Cross-sectional study	Military for vaccination in hospital (NR)	Military N = 226	2014–2015: 8.0	Adults (19–28 y): 8.0	All: 8.0 - A: 1.8 - C: 4.0 - W135: 0.4 - Y: 1.3 - Other: 0.4
Iran [35]	Longitudinal study	Military training centre (south-east of Iran)	Military N = 764 (1st sample) N = 692 (2nd sample)	1st sample 2002–2003: 11.4	1st sample 2002–2003 Adults (18–≥ 20 y): 11.4	1st sample 2002–2003 All: 11.4
				2nd sample 2002–2003: 32.9	2nd sample 2002–2003 Adults (18–≥ 20 y): 32.9	2nd sample 2002–2003 All: 32.9
Iran [36]	Longitudinal study	Waiting hall of the airport before and after the Hajj (Zahedan)	Pilgrims N = 422 (before Hajj 2012) N = 422 (post Hajj 2012) Hajj study	Before Hajj 2012: 0	Before Hajj Adults (21–95 y): 0	Before Hajj All: 0
				Post Hajj 2012: 1.4	Post Hajj Adults (21–95 y): 1.4	Post Hajj All: 1.4
Iran [37]	Cross-sectional study	3 dormitories affiliated with Kerman University of Medical Sciences (Kerman)	University students N = 335	2017–2018: 6.9	Adults (18–34 y): 6.9	All: 6.9 - C: 3.0 - Other: 3.9
Iran [38]	Cross-sectional study	16 schools (Kashan)	Students N = 115	2011–2012: 6.0–12.3	Children (7–14 y): 6.0–7.1	All: 8.9 - B: 0.6 - C: 8.3
					Children and adults (7–19 y): 8.9–12.3	
Kuwait [39]	Cross-sectional study	Pilgrims who attended a post-Hajj gathering (NR)	Pilgrims N = 177 Hajj study	2005: 0.0	Adults (NR): 0.0	All: 0.0
Saudi Arabia [43]	Longitudinal study	King Khalid National Guard Hospital (Mecca/Jeddah area)	Pilgrims and hospital personnel N = 190 (before Hajj 2001) N = 137 (post Hajj 2001) Hajj study	Before Hajj 2001: 7.4	Before Hajj 2001 Adults (18–61 y): 7.4	Before Hajj 2001 All: 7.4 - A: 0.5 - B: 1.1 - W135: 2.6 - Other: 0.5–2.6
				Post Hajj 2001: 0.7	Post Hajj 2001 Adults (18–61 y): 0.7	Post Hajj 2001 All: 0.7 - A: 0 - B: 0 - W135: 0.7 - Other: 0.0
Saudi Arabia [42]	Cross-sectional study	Mina Hospital outpatient clinics (Mina)	Pilgrims N = 344 Hajj study	2003: 1.6–4.3	Children (0–19y): 3.0	All: 1.6–4.3 - B: 0.3 - W135: 0.6 - Other: 2.3
					Adults (20–> 40 y): 1.6–4.3	
					Children and adults (0–> 40 y): 3.2	
Saudi Arabia [44]	Cohort study	Arriving pilgrims: Hajj Terminal of the King Abdul Aziz International Airport (Jeddah); departing pilgrims: tents for Hajj pilgrims (Mina)	Pilgrims N = 1055 (unpaired arriving pilgrims 2014) N = 373 (unpaired departing pilgrims 2014) N = 628 (paired cohort arriving and departing pilgrims 2014) Hajj study	I. Unpaired arriving pilgrims 2014: 3.4	I. Adults (18–> 65 y): 3.4	I. All: 3.4 - B: 2.3 - Other: 1.1
				II. Unpaired departing pilgrims 2014: 1.6	II. Adults (> 18–100 y): 1.6	II. All: 1.6 - B: 0.5 - Other: 1.1
				IIIa. Paired cohort arriving pilgrims 2014: 2.5	IIIa. Adults (18–> 65 y): 2.5	IIIa. All: 2.5 - B: 2.2
				IIIb. Paired cohort departing pilgrims 2014: 1.3	IIIb. Adults (18–> 65 y): 1.3	IIIb. All: 1.3 - B: 1.3
Saudi Arabia [40]	Cross-sectional study	King Abdul Aziz International Airport, King Khalid International Airport, camping (Jeddah Riyadh, Mina)	Pilgrims N = 715 (before Hajj) N = 745 (post Hajj) Hajj study	Before Hajj 2000: 8.0	Before Hajj 2000 Adults (18–100 y): 8.0	Before Hajj 2000 All: 8.0 - A: 0.4 - B: 1.3 - W135: 0.4 - Y: 0.6 - Other: 5.2
				Post Hajj 2000: 10.4	Post Hajj 2000 Adults (18–100 y): 10.4	Post Hajj 2000: 10.4 - A: 0.0 - B: 0.4 - W135: 4.6 - Y: 0.0 - Other: 5.2
Saudi Arabia [41]	Longitudinal study	King Abdul-Aziz International Airport (Jeddah)	Pilgrims Umra visitors N = 979 (before Umra) N = 979 (post Umra) Hajj N = 1433 (before Hajj) N = 613 (post Hajj) Hajj study	Before Umra 2008: 2.5	Before Umra 2008 Adults (18–100 y): 2.5	Before Umra 2008 All: 2.5
				Post Umra 2008: 5.7	Post Umra 2008 Adults (18–100 y): 5.7	Post Umra 2008 All: 5.7
				Before Umra 2008: 5.9	Before Umra 2008 Adults (18–100 y): 5.9	Before Umra 2008 All: 5.9
				Post Hajj 2008: 11.1	Post Hajj 2008 Adults (18–100 y): 11.1	Post Hajj 2008 All: 11.1
Saudi Arabia [45]	Cross-sectional study	Hajj terminal of King Abdulaziz International Airport (Jeddah)	Pilgrims N = 2249 (post Hajj) Hajj study	Post-Hajj 2017: 4.6	Children and adults (11–100 y): 4.6	All: 4.6 - A: 0.1 - B: 0.4 - C: 0.4 - W135: 0.1 - X: 0.3 - Y: 0.1
Turkey [46]	Cross-sectional study	Military unit (NR)	Military N = 1995	2008: 4.2	Adults (21–29 y): 4.2	All: 4.2 - A: 0.1 - B: 0.3 - C: 0.4 - W135: 0.5 - Y: 0.7 - Other: 2.3
Turkey [57]	Longitudinal study	Hacettepe University (Ankara)	Pilgrims and household contacts N = 472 (pilgrims before Hajj 2010) N = 296 (pilgrims post Hajj 2010) N = 39 (household contacts 2–3 months post Hajj 2010) Hajj study	I. Pilgrims before Hajj 2010: 13.3	I. Children and adults (15–64 y): 13.3	I. All: 13.3 - A: 0.2 - B: 1.9 - W135: 11.0 - Y: 0.2
				II. Pilgrims post Hajj 2010: 27.4	II. Children and adults (15–64 y): 27.4	II. All: 27.4 - A: 0.3 - B: 1.7 - W135: 25.0 - Y: 0.3
				III. Household contacts post Hajj 2010: 25.6	Children and adults (NR): 25.6	All: 25.6
						W135: 25.6
					Adults (20–70 y): 2.5	
Turkey [122]	Cohort study	Esenboğa Airport (Ankara)	Pilgrims N = 229 (pre-Hajj, paired cohort) N = 229 (post-Hajj, paired cohort) Hajj study	I. Pre-Hajj 2018: 3.9	I. Children and adults (10–80 y): 3.9	I. All: 3.9 - B: 3.9
				II. Post Hajj 2018: 0.4	II. Children and adults (10–80 y): 0.4	II. All: 0.4 - B: 0.4

Data on asymptomatic meningococcal carriage in the general population were available for Turkey only (n = 6 studies), spanning a time period of 2000–2018 [27–32]. As data were only available for one country, no overall conclusions can be drawn for the EM region as a whole. Asymptomatic carriage rates were 1.2 [27]–11.0% [31] in children (n = 4 studies), 1.3 [32]–10.3% [31] in adults (n = 3 studies) and 0.6 [29]–6.3% [31] in a mixed group of children/adults (n = 2 studies). Age ranges for children-only groups varied by study (0–10 years [27], 7–14 years [28], 0–14 years [30] and 10–17 years) [31].

For the risk groups (e.g. household contacts, military personnel, pilgrims or students), the most extensive data were available in the pilgrim population (Table 1). Data were available from Iran (n = 6) [33–38], Kuwait (n = 1) [39], Saudi Arabia (n = 6) [40–45], and Turkey (n = 3) [46–48]. Asymptomatic carriage rates ranged from 4.2% (Turkey) [46] to 32.9% (Iran) [35] in the military risk group (n = 3 studies), from 0.0% (Iran [36], Kuwait [39]) to 27.4% (Turkey [47]) in the pilgrim risk group (n = 11 studies) and from 6.9 to 12.3% (both Iran [37, 38]) in the student risk group (n = 2 studies). One data point was available for household contacts, showing asymptomatic carriage at 25.6% (Turkey) [47].

## Objective 2: IMD epidemiology

### Incidence

In the EM region, IMD incidence data were identified for Bahrain (n = 1) [49], Iran (n = 1) [50], Kuwait (n = 1) [51], Qatar (n = 1) [52], Saudi Arabia (n = 4) [53–56], Turkey (n = 3) [57–59], and Yemen (n = 1) [60]. For the NA region, IMD incidence data were available for Egypt (n = 2) [61, 62], Morocco (n = 1) [63], and Tunisia (n = 1) [64]. Age ranges were not clearly defined in many studies (e.g. children and adults [0–100 years]; Table 2).

**Table 2:** Incidence of meningococcal disease

References	Study design	Setting (region)	Population Total number of cases	Incidence (/100,000)		
				Year	Age category (age range)	Serogroup
EM region						
Bahrain [49]	Surveillance study	National surveillance system of communicable diseases (Whole country)	General population N = NR	1990: 0.83	Children and adults (0–100 y): 0.83	All: 0.83
Iran [50]	Cross-sectional study	Data bank at the Center for Disease Control Iran (Whole country)	General population N = 1370	2000: 0.67	Children and adults (0–100 y): 0.67–1.22	All: 0.67–1.22
				2001: 1.22		
				2002: 1.01		
				2003: 0.73		
				2004: 0.86		
Kuwait [51]	Surveillance study	All six general hospitals and other subspecialty hospitals (Whole country)	General population N = 293	1987–2013: 0.5	Children and adults (0–100 y): 0.13–0.74	All: 0.13–0.74
				1987–1997: 0.74		
				1998–2008: 0.5		
				2009–2013: 0.13		
Qatar [52]	Cross-sectional study	Hamad Medical Corporation (Doha)	General population N = 2	1998–2000: 0.12	Children and adults (0–100 y): 0.12	All: 0.12
Saudi Arabia [53]	Surveillance study	Hospitals with paediatrics services from 5 cities (Al-Jouf, Buraidah, Gateef, Al-Baha, Medina)	General population N = 37	1999–2001: 0.0–19.7	Children (0–5 y): 0.0–19.7	All: 0.0–19.7
Saudi Arabia [55]	Surveillance study	Laboratories reports from all 20 health regions (Whole country)	General population N = 1103	1995–2011: 0.2	Children and adults (0–100 y): 0.2	All: 0.2
Saudi Arabia [56]	Cross-sectional study	Saudi Aramco Medical Services Organization (Whole country)	General population N = 10	1993–1999: 1.9	Children and adults (0–100 y): 1.9–2.0	All: 1.9–2.0
				2000–2005: 2.0		
Turkey [58]	Surveillance study	12 paediatric hospitals and clinics (7 regions in Turkey: Central Anatolia, Marmara, South East Anatolia, Aegean, East Anatolia, Mediterranean, Black Sea)	General population N = 85	2013: 0.3	Children (0–17 y): 0.3–0.9	All: 0.3–0.9
				2014: 0.9		
Turkey [57]	Surveillance study	12 paediatric hospitals and clinics (7 regions in Turkey: Central Anatolia, Marmara, South East Anatolia, Aegean, East Anatolia, Mediterranean, Black Sea)	General population N = 333	2005–2006: 1.9	Children (0–17 y): 0.5–1.9	All: 0.5–1.9
				2007–2008: 1.5		
				2009–2010: 0.5		
				2011–2012: 0.6		
Turkey [59]	Surveillance study	27 hospitals located in seven regions of Turkey and represented 45 of the population	General population N = 89	2015–2016: 0.3	Children (0–18 y): 0.3–0.4	All: 0.3–0.4
				2017–2018: 0.4		
Yemen [60]	Cross-sectional study	Al-Salam hospital (Sana’a)	General population N = 81	2000: 8.8–20.5	Children (0–15 y): 8.8–20.5	All: 8.8–20.5
Saudi Arabia [54]	Surveillance study	Records (Ministry of Health and regional health directorates), clinical laboratory records and inpatient charts (hospitals) (Mecca, Medina, Jeddah)	Pilgrims N = 253 Hajj study, outbreak study	January–June 2000: 5.8–8.9	Children and adults (0–80 y): 5.8–8.9	A: 5.8
						W135: 8.9
NA region						
Egypt [61]	Surveillance study	MOHP (Whole country)	General population N = NR	1967–1992: 0.7–2.0 1992–2003: 0.1	Children and adults (0–100 y): 0.1–2.0	All: 0.1–2.0
Egypt [62]	Cross-sectional study	Communicable Disease Hospital (Alexandria)	General population N = 314	1997–2006: 1.06	Children and adults (0–100 y): 1.06	All: 1.06
Morocco [63]	Surveillance study	Epidemiology of the Health Systems Networks Service (Kenitra delegation)	General population N = 29	2014–2018: 2.96	Children and adults (0–100 y): 2.11–3.75	All: 2.11–3.75
				2014: 3.59		
				2015: 2.79		
				2016: 2.11		
				2017: 2.53		
				2018: 3.75		
Tunisia [64]	Cross-sectional study	Bechir Hamza Children’s Hospital (Tunis)	General population N = 73	2014: 1.5	Children (0–18 y): 1.5	All: 1.5

Studies in the EM region included data collected between 1987 and 2018. The majority of studies did not differentiate incidence by serogroup. IMD incidence in the general population ranged from 0 to 19.7/100,000 in children aged between 0 and 5 years in Saudi Arabia (Medina) in 1999–2001 [53], and from 8.8 to 20.5/100,000 in children aged between 0 and 5 years in Yemen (Sana’a) in 2000 [60]. In the remaining studies reporting on incidence in children in the general population, all were from Turkey. Incidence ranged from 0.3/100,000 up to 0.9/100,000 [58, 59] between 2013 and 2016 in two studies, and fell from 1.9/100,000 in 2005–2006 to 0.6/100,000 in 2014 in another study [57]. The incidence of IMD in children and adults in the general population across the EM region ranged from 0.12/100,000 (Qatar; 2002) [52] to 2.0/100,000 (Saudi Arabia) [56] between 1987 and 2013 [49–52, 55, 56]. Incidence was 0.6/100,000 [50] in a military population in Iran (2000–2004) and ranged from 5.8 to 8.9/100,000 in a study of pilgrims in Saudi Arabia in 2000 [54].

For the NA region, studies included incidence data collected between 1967 and 2018, all in the general population. IMD incidence in children only (aged between 0 and 18 years) was reported at 1.5/100,000 [64] in one study in Tunisia in 2014. Data for children and adults was available from two Egyptian studies and one Moroccan study, with incidence ranging from 0.1/100,000 [61] to 3.75/100,000 [63]. Data from the grey literature included a report from the Ministry of Health of the Kingdom of Saudi Arabia. This source reported an overall incidence of 0.02–0.03/100,000 persons in 2017, rising very slightly from 0.01 in 2013 [65]. A World Health Organization (WHO) consultation from 2001 also reported on the number of meningococcal disease cases in Saudi Arabia over time, with the number of annual cases rising from < 20 in 1995 to 253 in 2000 [66].

### Serogroup distribution

There were more studies available on serogroup distribution from the EM region versus the NA region. Data were available from Iran (n = 2) [67, 68], Kuwait (n = 1) [51], Qatar (n = 1) [69], Saudi Arabia (n = 6) [54–56, 70–72], and Turkey (n = 8) [57–59, 73–77], in the EM region (spanning 1987–2018), and from Egypt (n = 3) [61, 78, 79], Morocco (n = 6) [80–85], and Tunisia (n = 6) [64, 86–90], in the NA region (spanning 1977–2019). Serogroups A, B and W were most frequently reported in the EM region, while B and C serogroups were most frequently reported in the NA region.

### CFR data

**Fig. 2:**
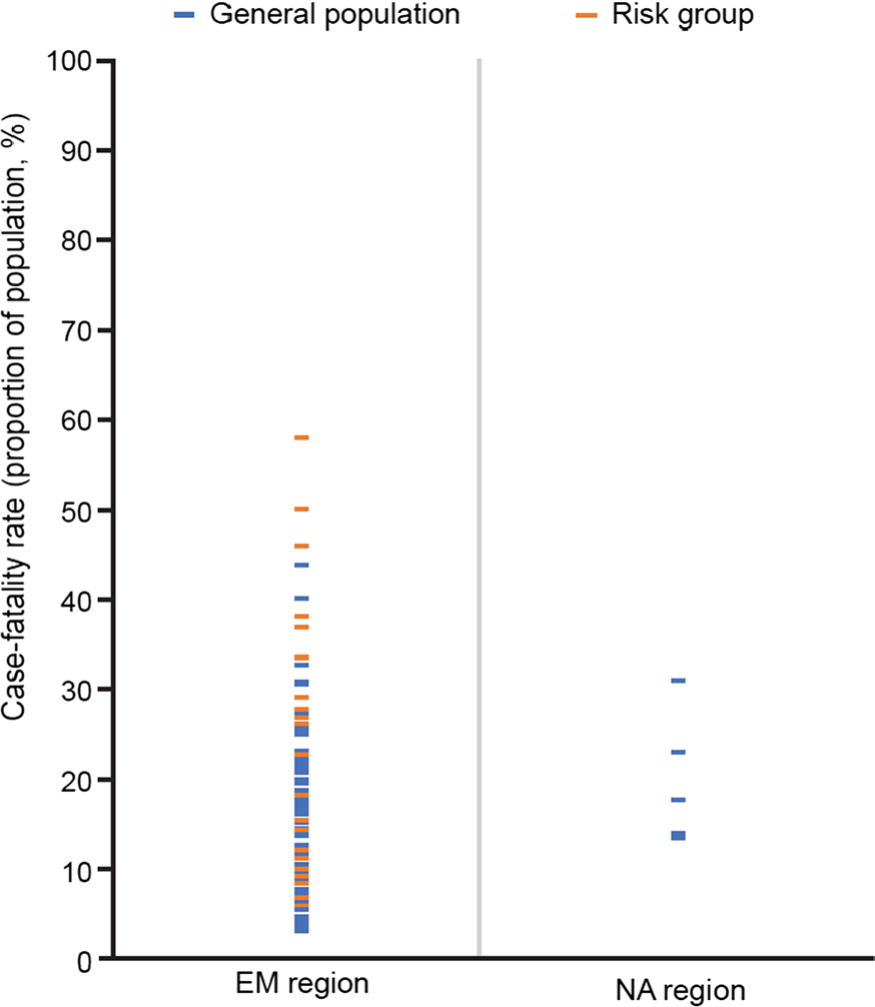
Reported case-fatality rate (general population and risk groups). *EM* Eastern Mediterranean region, *NA* North Africa region. Countries providing data for EM region: Iran, Kuwait, Pakistan, Qatar, Saudi Arabia, Turkey and Yemen. Countries providing data for NA region: Egypt and Tunisia

CFR data were available in 27 studies in the EM region (Iran n = 4, [50, 67, 91, 92]; Kuwait n = 3, [51, 93, 94]; Pakistan n = 2, [95, 96]; Qatar n = 2, [52, 69]; Saudi Arabia n = 4, [53–55, 71]; Turkey n = 10, [57–59, 74, 76, 97–101]; and Yemen n = 2, [60, 102]) and five studies in the NA region (Egypt n = 3, [62, 78, 79]; Morocco n = 1, [63] and Tunisia n = 1, [89]); one multi-country study spanned both regions [103]. Data were collected between 1987 and 2018. Data on sample sizes for each study can be found in Additional file 1: Table S1. CFRs in the general population were very heterogeneous across the EM region; however, the majority were below 50% (Fig. 2). Based on the more limited dataset from the NA region (n = 5; studies predominantly based in Egypt), rates ranged from 13.4% (Egypt [62]) to 31.0% (Morocco [63]) between 1997 and 2018. Reported CFRs in the EM region were generally lower than 25% in children in the general population, with only one study reporting a CFR value greater than 25% (43.8%; Turkey [101]). CFRs varied more widely in adults in the general population, with the majority falling between approximately 5% and 50%. In the NA region, the CFR ranged from 17.7% [89] to 23.0% [79] in children and from 13.4% [62] to 31.0% [63] in mixed populations of children and adults. Data from the multi-country study reported CFRs ranging from 5.7% to 6.2% between 2004 and 2010 in the general population (5.7% in children only [aged 0–5 years] and 5.9–6.2% in children and adults) [103].

Four studies in the EM region provided data on CFRs in risk groups (military n = 1 [50] and pilgrims n = 3 [54, 55, 71]). CFR data from the military population (based in Iran) ranged from 0 to 50.0% between 2000 and 2004, [50] while data from the pilgrim population (all studies in Saudi Arabia) showed a CFR range from 0.0% [55, 71] to 57.9% [71]. Data from the previously-mentioned WHO consultation reported a CFR of 27.7% among IMD cases in Saudi Arabia in 2001, with a CFR of 32.1% observed in the subset of cases associated with the Hajj [66].

## Objective 3: IMD presentation and management

### Clinical presentation

Five studies in the EM region (Iran n = 1 [91]; Saudi Arabia n = 1 [71]; and Turkey n = 3 [97, 100, 101]) and two studies in the NA region (Morocco n = 1 [104] and Tunisia n = 1 [89]) had data on clinical presentation. The majority of studies were in children aged 0–14 years (n = 6), and the study in Saudi Arabia was in adults aged 18–80 years [71]. The most common presentations across both regions were meningitis (12.5% [101]–63.8% [71]), followed by meningococcaemia (7.6% [71]–56.3% [101]), and meningitis plus meningococcaemia (11.4% [104]–36.2% [71]).

### Hospitalisation

Six studies in the EM region (Kuwait n = 1 [94]; Pakistan n = 1 [105]; Saudi Arabia n = 2 [53, 54]; and Turkey n = 2 [97, 101]) and two studies in the NA region (Egypt n = 2 [62, 78]) included data on hospitalisation. In the EM region studies in children, the mean/median length of hospital stay (LOS) ranged from 9.8 [101] to 21.9 [97] days. Intensive care unit (ICU) admission was required for 30.0% of cases in Kuwait [94] and 5.4% of cases in Saudi Arabia [53], and mean ICU LOS ranged from 5.0 [53] to 7.8 days [97]. In children and adults in Saudi Arabia, 24.1% required ICU admission, with 64.7% of this group requiring ICU care for more than 1 day [54]. Studies from Egypt (in children and adults) reported that LOS was generally lower than 15 days (median LOS 10 days [78], mean LOS 14.7 days [62]).

### Symptoms, complications and sequelae

Data were collected on acute symptoms, admission and acute complications, and post-discharge complications and sequelae; full information is provided in Table 3. Since outcomes were not well defined in the majority of included articles, it was not possible to provide a list of definitions for these outcomes.

**Table 3:** Symptoms, complications and sequelae

a) Acute symptoms								
Region	EM						NA	
Country	Iran [106]	Iran [91]	Qatar [69]	Turkey [97]	Turkey [107]	Saudi Arabia [71]	Egypt [78]^b^	Morocco [104]
Population type	GP	GP	GP	GP	GP	P	GP	GP
Number of patients in study	3	68	25	65	7	105	203	35
Date range, year	2009–2011	1992–2002	1992–2008	2000–2005	1995–2003	February–April 2000	1998–2004	2011–2013
Age range, year	Children (0.5–10)	Children (0–13)	Children, adults (0–68)	Children (0.5–10)	Children, adults (15–60)	Adults (18–80)	Children, adults (0–75)	Children (0–13)
Symptoms								
Proportion of patients experiencing acute symptoms (%)	0.0–100	8.8–64.7	4.0–80.0	7.7–95.4	71.4	28.6	16.0–79.0	5.7–65.7
Stiff neck	100	55.9	52.0				79	
Rash	0	64.7	28.0		71.4	28.6	16	65.7
Fever	66.7		80.0	95.4				
Meningeal irritation syndrome^a^	66.7							
Seizure	66.7	8.8	4.0–12.0	7.7				11.4
Headache		55.9	48.0				66.0	31.4
Vomiting			56.0					40.0
Altered consciousness			48.0	27.7				
Diarrhoea				9.2				11.4
Purpura				92.3				
Photophobia							37	
Neck pain								17.1
Abdominal pain								5.7
Lethargy								48.6
Irritability								8.6

b) Complications at admission and acute complications			
Region	EM		NA
Reference	Iran [91]	Turkey [97]	Morocco [104]
Population type	GP	GP	GP
Number of patients in study	68	65	35
Date range, year	1992–2002	2000–2005	2011–2013
Age range, year	Children (0–13)	Children (0.5–10)	Children (0–13)
Complications at admission and acute complications			
Proportion of patients experiencing complications at admission and acute complications (%)	2.9–38.2	3.1–38.5	11.4–54.3
Septic shock	38.2	38.5	
Conjunctivitis	2.9		
Pericarditis	2.9		
Myocarditis		4.6	
Purpura fulminans		4.6	
Acute renal failure		3.1	
ARDS		3.1	
Arthralgia			11.4
Hemodynamic disorder/sepsis			54.3

c) Post-discharge complications/sequelae										
Region	EM								NA	
Reference	Kuwait [93]	Kuwait [94]	Qatar [69]	Qatar [52]	Saudi Arabia [124]	Saudi Arabia [71]	Turkey [98]	Turkey [99]	Egypt [78]	Tunisia [89]
Population type	GP	GP	GP	GP	GP	P	GP	GP	GP	GP
Number of patients in study	30	10	25	2	37	105	2	15	203	79
Date range, year	2001–2003	2010–2014	1992–2008	1998–2000	1999–2001	February–April 2000	1994–2004	2012–2016	1998–2004	1997–2006
Age range, year	Children (0–12)	Children (0–12)	Children, adults (0–68)	Children, adults (0–100)	Children (0–5)	Adults (18–80)	Children (2–4)	Children (0–18)	Children, adults (0–75)	Children (0–13)
Post-discharge complications and sequelae										
Proportion of patients experiencing post-discharge complications/sequelae (%)	3.4	0	4.0–24.0	0	2.7	1	50	33.3	5	1.3–7.6
Hearing impairment	3.4									3.8
Motor palsy	3.4									
Aphasia			8.0							
Limb weakness			8.0							
Focal findings			16.0							
Cranial nerve palsy			4.0					33.3		
Amputation							50.0			1.3
Neurological complication									5.0	
Skin necrosis										7.6
Non-specific sequelae		0.0	24.0	0.0	2.7	1.0				

All studies are cross-sectional unless otherwise stated

*ARDS* acute respiratory distress syndrome, *EM* Eastern Mediterranean region, *GP* general population, *NA* North Africa region, *P* pilgrims

^a^Surveillance study

^b^Meningeal irritation syndrome encompasses Kernig’s sign and Brudzinski’s sign [123]

Six studies from the EM region (Iran n = 2 [91, 106]; Qatar n = 1 [69]; Saudi Arabia n = 1 [71]; and Turkey n = 2 [97, 107]) and two studies from the NA region (Egypt n = 1 [78] and Morocco n = 1 [104]) included information on acute symptoms of meningococcal disease. Commonly observed symptoms across both regions included rash, seizure, headache, stiff neck and fever. No particular pattern was observed for symptoms or age groups. Serogroup was infrequently reported in these studies and so it was not possible to correlate serogroups with symptoms. Data on complications at admission and acute complications were available from three studies (all in children), of which two were in the EM region (Iran n = 1 [91]; Turkey n = 1 [97]), and one in the NA region (Morocco n = 1 [104]). Septic shock was reported in approximately 40% of patients in the studies from the EM region, while hemodynamic disorder/sepsis were reported in over half of patients in the study from the NA region. Data on post-discharge complications and sequelae were available from eight EM region studies (Kuwait n = 2 [93, 94]; Qatar n = 2 [52, 69]; Saudi Arabia n = 2 [53, 71]; and Turkey n = 2 [98, 99]) and two NA region studies (Egypt n = 1 [78] and Tunisia n = 1 [89]). The majority of studies in the EM region reported non-specified sequelae; there was little overlap across studies in terms of specific reported events for either region.

### Antibiotic treatment

Three studies in Turkey and one in Qatar had data on antibiotic treatment; no studies were retrieved from the NA region. The Turkish studies focused on antibiotic use in children only [74, 97, 100], while the Qatari study included data from a mixed population of children and adults aged 0–80 years [69]. Ceftriaxone monotherapy was prescribed to 53.8 [97]–100% [100] of patients in all studies in Turkey, and in combination with vancomycin in one study (40.0% of cases) [74]. Penicillin was used by 9.8% of patients in another study [97]. In the Qatari study, ceftriaxone was prescribed in 80.0%, and penicillin in 20.0% of cases [69].

### Prophylaxis or vaccination of persons in close contact

No studies in the EM or NA region reported on prophylaxis or vaccination of persons in close contact with a known case of meningococcal infection.

## Discussion

This systematic review identified that high-risk groups such as military personnel, pilgrims and students remain a significant reservoir of asymptomatic meningococcal carriage in the EMNA region, with few studies specifying asymptomatic carriage by age group. In general, more studies were retrieved from the EM region compared with the NA region. Incidence of IMD in the EMNA region was poorly defined, with data only available for 7/15 countries in the EM region and 3/5 countries in the NA region. CFRs associated with IMD were heterogenous, and few data were available on CFRs in key risk groups (including household contacts, military personnel, pilgrims and students). Data were also heterogenous for complications and sequelae. Approximately one-third of patients were admitted to the ICU, and LOS was generally below 3 weeks (in hospital or in the ICU). Relatively few studies reported data on antibiotic treatment, which were reported in the EM region only. In studies that did provide data on antibiotic use, ceftriaxone was commonly used. Information on antibiotic resistance was not captured in this study. Furthermore, no studies in the EMNA region reported on prophylaxis or vaccination of persons in contact with known cases of infection, or on economic impact and evaluation. These data gaps indicate a pressing need for more studies from the region, ideally focusing on similar study outcomes for ease of comparison.

Results from a recent meta-analysis (spanning 2012–2017) indicate that *N. meningitidis* accounts for 9% and 36% of all bacterial meningitis cases in Eastern Mediterranean and African regions, respectively [108]. Furthermore, the burden of IMD in the Eastern Mediterranean Regional Office (EMRO) region is second highest after the African meningitis belt (bacterial meningitis is also second highest in the EMRO region compared with the African region), with several countries (where data exists) reporting moderate/high endemic rates [45, 109, 110]. In our review, the most comprehensive IMD incidence data were available for Saudi Arabia. There were no EM regional IMD incidence data from Bahrain, Iraq, Jordan, Lebanon, Oman, Palestinian Territories, Syria, Turkey, and the United Arab Emirates.

It is interesting to compare our findings with those from other regions. Data from various sources showed that the overall incidence of IMD was 0.6/100000 population in Europe (in 2017), 0.13/< 1.0 per 100,000 population in the USA/Canada (2013/2015), 0.16–0.8/100000 population in Latin America (2012–2018), 1.5/100000 population in Australia (2013–2017), 1.8/100000 population in China (2000–2010), < 1.0/100000 population in Asia (2020; excluding China) and < 0.2/100000 population overall in the Asia–Pacific region (2020) [17, 111–114]. Incidence in the EM region in the current study ranged from 0 to 20.5/100,000 persons (based on data from seven countries). In contrast, incidence in the NA region ranged from 0.1 to 3.75/100000 persons (based on data from Egypt, Morocco and Tunisia). The incidence of IMD was highest in infants and young children in several regions (e.g. Europe, USA/Canada, Latin America) which is similar to data from EMNA presented here [112].

IMD CFRs are often high in the EMNA region with rates up to 50% reported, and many studies reporting rates between 5 and 25%. It is possible that the high CFRs observed in the EMNA region could potentially reflect inadequate or delayed treatment [1]. CFRs elsewhere vary, both by region and by age group. For example, overall CFR was 10% in Europe in 2017 and increased to 18% in cases in people aged 65 years and over [17]. Other countries also report high CFRs (e.g. China [33% between 2000 and 2010] and Brazil [20.7% between 2005 and 2011]) [111, 114].

Globally, the IMD CFR can be higher in infants than older children, but is highest in those ≥ 65 years of age [15]. Although data on CFRs in children and adults were identified in the EM and NA regions, it is difficult to make comparisons between age groups, especially when considering heterogeneity in study design. Furthermore, given the importance of age for the epidemiology of IMD, it is notable that patients were not routinely separated by age in the studies included here.

In our study, serogroups A, B and W were common in the EM region, and the B and C serogroups were common in the NA region. This is in line with findings from a recent systematic review, which reported that *N. meningitidis* serogroup B is the predominant cause of IMD in various parts of Europe, North America, Latin America and the Western Pacific, while serogroups C and W were responsible for a substantial proportion of IMD cases in large parts of Africa and Latin America. It should also be noted that serogroups W, A and X were reported as causes of IMD in many countries in the African meningitis belt [115, 116].

The WHO has also identified the need for awareness of meningococcal disease burden and development of surveillance systems to characterise national epidemiology in all countries [14, 117]. In particular, the WHO global roadmap for defeating meningitis by 2030 recommends that all countries should design and implement a surveillance system which allows integration of public/private healthcare and also covers key components such as epidemiology, laboratory and data management [109]. Evaluation of mass vaccine programmes in countries in the African meningitis belt is also recommended [117]. Introduction of vaccines specific to particular serogroups has previously led to a reduction in the burden associated with those serogroups in the African meningitis belt and Europe [17, 112, 118], illustrating that vaccination programmes may change the burden and molecular epidemiology of IMD. However, migration dynamics may also influence IMD burden and epidemiology in the EMNA region, due to the increased risk of IMD transmission during the Umrah and Hajj pilgrimages [11, 13, 14, 20]. These dynamics render the regional situation even more complex, and provide at least a partial explanation as to why the WHO goals of increased awareness/improved surveillance systems are still far from being met in the EMNA region.

It has long been known that vaccination has important benefits in terms of protecting individuals against IMD, lowering the carriage rates of *N. meningitidis* in the community and providing herd protection for non-vaccinated individuals [119]. Since the 2000s, these Hajj-related outbreaks have been reduced through local vaccination programmes for pilgrims with the quadrivalent meningococcal vaccine in Saudi Arabia [13]. Saudi Arabia also now demands proof of recent meningococcal vaccination (specifying a quadrivalent vaccine [ACYW]) as a visa requirement for international pilgrims travelling to these gatherings [11, 120]. However, IMD burden in the region remains high and so further action is needed.

These data highlight gaps in our current understanding of the epidemiology and disease burden of IMD in the EMNA region [24]. This situation contrasts with the much stronger IMD surveillance data available for areas such as Europe [17] and the African meningitis belt [24], and may be linked to lack of systems or resources for disease surveillance within the EMNA region. It should be noted that the general lack of effective surveillance in the EMNA region occurs even though, as in Europe, IMD is a notifiable disease. To improve surveillance in this region, the WHO has funded the Invasive Bacterial Vaccine Preventable Diseases Laboratory Network, including a regional reference laboratory in Egypt and a site laboratory in Yemen; however, other EMNA countries in this study do not appear to have laboratories associated with this network [121]. In addition, despite some EMNA countries having national surveillance networks and laboratories with the ability to serogroup IMD samples [24, 115], there is still a general lack of published epidemiological studies from these countries. These findings suggest the need for a standardised global approach to IMD reporting to improve the epidemiological evidence base and address the high disease burden.

The strengths of this systematic literature review include that it was based on broad search terms and included articles in all languages and reports on data amassed over an 18-year period. Limitations include the lack of consistent data availability. In addition, the heterogeneity of the design/methodology of the selected studies and the resulting data means that it is difficult to compare outcomes across studies. As a result, the findings from this search may not reflect the true disease burden and serogroup distribution; therefore, further data on the epidemiology and disease burden of IMD in the EMNA region are needed.

In conclusion, our systematic review identifies the crucial need to increase national surveillance systems and laboratory capacity in this region in order to improve the quality of data and reporting. There is also a pressing requirement for conducting further research on the consequences and burden of meningitis and meningococcaemia. This will allow us to better understand their epidemiology and design health policies to reduce the subsequent burden on regional healthcare systems.

## Supplementary Information

**Additional file 1.** Additional information on methodology and data per region/country. PRISMA statement, details of systematic review strategy, total number of cases per study and associated CFR, Countries with/without data for each objective.

## Data Availability

The datasets used and/or analysed during the current study are available from the corresponding author on reasonable request.

## Abbreviations

AMEE: Asia, Middle East and Eurasia
CFR: Case-fatality rate
EM: Eastern Mediterranean
EMRO: Eastern Mediterranean Regional Office
HTA: Health technology assessment
ICU: Intensive care unit
IMD: Invasive meningococcal disease
LOS: Length of stay
MACV: Meningococcal A conjugate vaccine
NA: North Africa
PRISMA: Preferred Reporting Items for Systematic Reviews and Meta-Analyses
WHO: World Health Organization
