# Correction to: Systematic review of invasive meningococcal disease epidemiology in the Eastern Mediterranean and North Africa region

**DOI:** 10.1186/s12879-021-06844-8

**Published:** 2021-11-15

**Authors:** Alp Giray Dogu, Anouk M. Oordt-Speets, Femke van Kessel-de Bruijn, Mehmet Ceyhan, Amine Amiche

**Affiliations:** 1 Sanof Pasteur, Dubai, UAE; 2 Pallas, Rotterdam, The Netherlands; 3 Faculty of Medicine, Hacettepe University, Ankara, Turkey

## Correction to: BMC Infect Dis (2021) 21:1088 https://doi.org/10.1186/s12879-021-06781-6

Following the publication of the original article [1], some errors were identified in the text and in Table 3a.

The footnotes are currently labeled as:

- a Surveillance study.
- b Meningeal irritation syndrome encompasses Kernig’s sign and Brudzinski’s sign.

The footnotes should be labeled as:

- A Meningeal irritation syndrome encompasses Kernig’s sign and Brudzinski’s sign
- B Surveillance study.

The original article [1] has been corrected.
