# Supplementary material for: Systematic review of invasive meningococcal disease epidemiology in the Eastern Mediterranean and North Africa region
Source: BMC Infect Dis. 2021 Oct 22;21:1088. doi: 10.1186/s12879-021-06781-6 (PMC8540099; doi:10.1186/s12879-021-06781-6)
Supplement: Supplementary file 1 — Additional file 1. Additional information on methodology and data per region/country. PRISMA statement, details of systematic review strategy, total number of cases per study and associated CFR, Countries with/without data for each objective. [file 12879_2021_6781_MOESM1_ESM.docx]

**Systematic review of invasive meningococcal disease epidemiology in the Eastern Mediterranean and North Africa Region**

Alp Giray Dogu^1^, Anouk M Oordt-Speets^2^, Femke van Kessel-de Bruijn^2^, Mehmet Ceyhan^3^ and Amine Amiche^1^

^1^Sanofi Pasteur, Dubai, UAE; ^2^Pallas, Rotterdam, the Netherlands; ^3^Faculty of Medicine, Hacettepe University, Ankara, Turkey

# Additional Information

## PRISMA statement

##

| **Section/topic** | **#** | **Checklist item** | **Reported on page #** |
| --- | --- | --- | --- |
| **TITLE** | | |  |
| Title | 1 | Identify the report as a systematic review, meta-analysis, or both. | 1 |
| **ABSTRACT** | | |  |
| Structured summary | 2 | Provide a structured summary including, as applicable: background; objectives; data sources; study eligibility criteria, participants, and interventions; study appraisal and synthesis methods; results; limitations; conclusions and implications of key findings; systematic review registration number. | 2–3 |
| **INTRODUCTION** | | |  |
| Rationale | 3 | Describe the rationale for the review in the context of what is already known. | 4–5 |
| Objectives | 4 | Provide an explicit statement of questions being addressed with reference to participants, interventions, comparisons, outcomes, and study design (PICOS). | 5–7 |
| **METHODS** | | |  |
| Protocol and registration | 5 | Indicate if a review protocol exists, if and where it can be accessed (e.g., Web address), and, if available, provide registration information including registration number. | 6; Additional file (section 1.2) pages 4–8 |
| Eligibility criteria | 6 | Specify study characteristics (e.g., PICOS, length of follow-up) and report characteristics (e.g., years considered, language, publication status) used as criteria for eligibility, giving rationale. | 6–7; Additional file (section 1.2) pages 4–8 |
| Information sources | 7 | Describe all information sources (e.g., databases with dates of coverage, contact with study authors to identify additional studies) in the search and date last searched. | 6, 39 (Figure 1); Additional file (section 1.2) pages 5–7 |
| Search | 8 | Present full electronic search strategy for at least one database, including any limits used, such that it could be repeated. | Additional file (section 1.2) pages 5–6 |
| Study selection | 9 | State the process for selecting studies (i.e., screening, eligibility, included in systematic review, and, if applicable, included in the meta-analysis). | 6–7, 39 (Figure 1); Additional file (section 1.2) pages 7–9 |
| Data collection process | 10 | Describe method of data extraction from reports (e.g., piloted forms, independently, in duplicate) and any processes for obtaining and confirming data from investigators. | 6–7; Additional file (section 1.2) pages 8–9 |
| Data items | 11 | List and define all variables for which data were sought (e.g., PICOS, funding sources) and any assumptions and simplifications made. | 6; Additional file (section 1.2) pages 4–7 |
| Risk of bias in individual studies | 12 | Describe methods used for assessing risk of bias of individual studies (including specification of whether this was done at the study or outcome level), and how this information is to be used in any data synthesis. | 7 |
| Summary measures | 13 | State the principal summary measures (e.g., risk ratio, difference in means). | 6; Additional file (section 1.3) page 10 |
| Synthesis of results | 14 | Describe the methods of handling data and combining results of studies, if done, including measures of consistency (e.g., I^2^) for each meta-analysis. | 6 |
| Risk of bias across studies | 15 | Specify any assessment of risk of bias that may affect the cumulative evidence (e.g., publication bias, selective reporting within studies). | 7 |
| Additional analyses | 16 | Describe methods of additional analyses (e.g., sensitivity or subgroup analyses, meta-regression), if done, indicating which were pre-specified. | Not relevant |

| **Section/topic** | **#** | **Checklist item** | **Reported on page #** |
| --- | --- | --- | --- |
| **RESULTS** | | |  |
| Study selection | 17 | Give numbers of studies screened, assessed for eligibility, and included in the review, with reasons for exclusions at each stage, ideally with a flow diagram. | 8, 39 (Figure 1) |
| Study characteristics | 18 | For each study, present characteristics for which data were extracted (e.g., study size, PICOS, follow-up period) and provide the citations. | 8–13, 21–26 (references 27–107); Additional file (Supplementary Figure 1) |
| Risk of bias within studies | 19 | Present data on risk of bias of each study and, if available, any outcome level assessment (see item 12). | Not relevant |
| Results of individual studies | 20 | For all outcomes considered (benefits or harms), present, for each study: (a) simple summary data for each intervention group (b) effect estimates and confidence intervals, ideally with a forest plot. | a) 8–13, 28–38 (Tables 1–3), 40 (Figure 2) b) Not done |
| Synthesis of results | 21 | Present results of each meta-analysis done, including confidence intervals and measures of consistency. | Not applicable |
| Risk of bias across studies | 22 | Present results of any assessment of risk of bias across studies (see Item 15). | Not relevant |
| Additional analysis | 23 | Give results of additional analyses, if done (e.g., sensitivity or subgroup analyses, meta-regression [see Item 16]). | Not relevant |
| **DISCUSSION** | | |  |
| Summary of evidence | 24 | Summarize the main findings including the strength of evidence for each main outcome; consider their relevance to key groups (e.g., healthcare providers, users, and policy makers). | 14–18 |
| Limitations | 25 | Discuss limitations at study and outcome level (e.g., risk of bias), and at review-level (e.g., incomplete retrieval of identified research, reporting bias). | 17 |
| Conclusions | 26 | Provide a general interpretation of the results in the context of other evidence, and implications for future research. | 14–18 |
| **FUNDING** | | |  |
| Funding | 27 | Describe sources of funding for the systematic review and other support (e.g., supply of data); role of funders for the systematic review. | 19 |

## Details of systematic review strategy

**Review questions**

The PICO (population-intervention-comparison-outcome) method was used to specify the review questions (see below).

| 1 | Asymptomatic carriage |
| --- | --- |
| P | Adults and children, asymptomatic carriage of meningococcal disease in the AMEE region |
| I | NA |
| C | NA |
| O | Outcome by age group  - Asymptomatic carriage over time  - Serogroup distribution of asymptomatic meningococcal carriage |
| 2 | Epidemiology |
| P | Adults and children with meningococcal disease in the healthy population or at-risk populations, such as immunocompromised, hajj pilgrims, young adults at university and travellers in the AMEE region |
| I | NA |
| C | NA |
| O | Outcomes by age group and serogroup:  - Serogroup distribution over time  - Incidence/number of cases over time  - CFR over time  - Description of complications and sequelae (type, frequency)  Surveillance systems:  - Description of surveillance systems in the AMEE region |
| 3 | Disease burden/HRQoL |
| P | Adults and children with meningococcal disease in the AMEE region |
| I | NA |
| C | NA |
| O | If relevant, outcomes by age group and serogroup:  - Description of clinical presentation (type and frequency)  - Hospitalisation/re-hospitalisation rates and length of stay  - Description of treatment (type and number of persons on certain treatment)  - Prophylaxis/vaccination of persons in contact (description of contacts, type, uptake)  - Impact of disease on HRQoL of both the patient and family/caregivers  - Costs of meningococcal disease |
| 4 | Economic evaluation |
| P | Adults and children with meningococcal disease in the AMEE region |
| I | Vaccination with ACWY meningococcal vaccine |
| C | No vaccination/vaccination with other meningococcal vaccine |
| O | Cost-effectiveness of vaccination |
| 5 | HTA recommendations |
| P | Adults and children with meningococcal disease in the AMEE region |
| I | Vaccination with meningococcal vaccine (conjugate/monovalent/quadrivalent/polysaccharide) |
| C | NA |
| O | Drivers of vaccination recommendations reported in HTA studies |

AMEE: Africa, Middle East and Eastern Europe; CFR: case fatality rate; HRQoL: health-related quality of life; HTA: health technology assessment; NA: not applicable.

**Literature searches**

***Peer-reviewed literature***

The review was a PubMed (MEDLINE) literature search complemented with a search in Embase.com.

The searches were built using the PICO-framework. However, because of the different objectives and outcomes of interest on meningococcal disease it was decided to keep the search as broad as possible and to create only a search string on ‘meningococcal disease’ in combination with a search string for the regions and countries of interest, to prevent missing relevant articles. The search strategy was as follows:

Search strings PubMed (MEDLINE)

In order to find relevant articles for the review objectives in PubMed (MEDLINE), Pallas made search strings for meningococcal disease and countries part of the AMEE region:

#1. Meningococcal disease

"Meningococcal Infections"[Mesh] OR "Meningitis, Meningococcal"[Mesh] OR meningococc*[tiab] OR meningitidis[tiab] OR ((meningitis[tiab] OR septicaemia[tiab] OR septicemia[tiab]) AND (neisseria[tiab] OR meningitidis[tiab]))

#2. Geographical scope: AMEE countries

(Asia*[tw] AND Europe*[tw]) OR Eurasia*[tw] OR "Middle East"[Mesh] OR Middle East*[tw] OR West Bank*[tw] OR Near East*[tw] OR Gaza Strip[tw] OR Bahrain*[tw] OR Bahrain*[ad] OR Iran*[tw] OR Iran*[ad] OR Iraq*[tw] OR Iraq*[ad] OR Israel*[tw] OR Israel*[ad] OR Jordan*[tw] OR Jordan*[ad] OR Kuwait*[tw] OR Kuwait*[ad] OR Lebanon*[tw] OR Lebanon*[ad] OR Lebanese*[tw] OR Lebanese*[ad] OR Oman*[tw] OR Oman*[ad] OR Palestin*[tw] OR Palestin*[ad] OR Palastin*[tw] OR Qatar*[tw] OR Qatar*[ad] OR Saudi*[tw] OR Saudi*[ad] OR KSA[tw] OR KSA[ad] OR Syria*[tw] OR Syria*[ad] OR Emirate*[tw] OR Emirate*[ad] OR UAE[tw] OR UAE[ad] OR Yemen*[tw] OR Yemen*[ad] OR Algeria*[tw] OR Algeria*[ad] OR Egypt*[tw] OR Egypt*[ad] OR Libya*[tw] OR Libya*[ad] OR Morocc*[tw] OR Morocc*[ad] OR Tunis*[tw] OR Tunis*[ad] OR Afghan*[tw] OR Afghan*[ad] OR Georgia*[tw] OR Georgia*[ad] OR Kazakh*[tw] OR Kazakh*[ad] OR Kirghiz*[tw] OR Kirghiz*[ad] OR Kyrgyz*[tw] OR Kyrgyz[ad] OR Pakistan*[tw] OR Pakistan*[ad] OR Russia*[tw] OR Russia*[ad] OR Turk*[tw] OR Turk*[ad] OR Ukrain*[tw] OR Ukrain*[ad] OR Uzbek*[tw] OR Uzbek*[ad]

Limit:

• Publication date: from 2000 onwards

The combination of the search strings (i.e. #1 AND #2) and the publication date limit yields 792 hits (dd. 08-02-2021).

Search strings Embase.com

In order to find relevant articles for the review objectives in Embase.com, Pallas made search strings for meningococcal disease and part of the AMEE region:

#1. Meningococcal disease

'meningococcosis'/exp OR 'epidemic meningitis'/exp OR meningococc*:ti,ab OR meningitidis:ti,ab OR ((meningitis:ti,ab OR septicaemia:ti,ab OR septicemia:ti,ab) AND (neisseria:ti,ab OR meningitidis:ti,ab))

#2. Geographical scope: AMEE countries

(Asia* AND Europe*) OR Eurasia* OR 'Middle East'/exp OR Middle East* OR West Bank* OR Near East* OR Gaza Strip OR Bahrain*:ti,ab,ad OR Iran*:ti,ab,ad OR Iraq*:ti,ab,ad OR Israel*:ti,ab,ad OR Jordan*:ti,ab,ad OR Kuwait*:ti,ab,ad OR Lebanon*:ti,ab,ad OR Lebanese*:ti,ab,ad OR Oman*:ti,ab,ad OR Palestin*:ti,ab,ad OR Palastin*:ti,ab,ad OR Qatar*:ti,ab,ad OR Saudi*:ti,ab,ad OR KSA:ti,ab,ad OR Syria*:ti,ab,ad OR Emirate*:ti,ab,ad OR UAE:ti,ab,ad OR Yemen*:ti,ab,ad OR Algeria*:ti,ab,ad OR Egypt*:ti,ab,ad OR Libya*:ti,ab,ad OR Morocc*:ti,ab,ad OR Tunis*:ti,ab,ad OR Afghan*:ti,ab,ad OR Georgia*:ti,ab,ad OR Kazakh*:ti,ab,ad OR Kirghiz*:ti,ab,ad OR Kyrgyz*:ti,ab,ad OR Pakistan*:ti,ab,ad OR Russia*:ti,ab,ad OR Turk*:ti,ab,ad OR Ukrain*:ti,ab,ad OR Uzbek*:ti,ab,ad

Limit:

• Publication date: from 2000 onwards

The combination of the search strings (i.e. #1 AND #2) and the publication date limit yields 953 hits (dd. 09-02-2021).

After removal of duplicates from the two literature databases in Endnote, 1182 unique hits remained:

Search results PubMed (MEDLINE) and Embase.com

| Database | Number of hits* |
| --- | --- |
| PubMed (MEDLINE) | 792 |
| Embase.com | 953 |
| Total number of hits | 1745 |
| Total number of hits - unduplicated | 1182 |

*Searches conducted February, 2021

In the search for articles that reported on costs of meningococcal disease, and HTA reports, the hits retrieved from PubMed (MEDLINE) and Embase.com were checked for the terms ‘cost’, ‘HTA’ or ‘health technology assessment’ in all indexed fields (e.g. author, title, journal, keywords, etc.) by the search function in Endnote. All retrieved hits were screened following the standard procedures outlined in 2.3 Selection procedure.

Additionally, a search for HTAs was performed in Google and Google Scholar using the term HTA/health technology assessment and meningococ*/meningococcal in April 2019. Not included in the search output in Google Scholar search were patents and citations, and articles published before 2000.

***Grey literature (i.e. hand search)***

To fill the gaps from the peer-reviewed literature, a grey literature search was conducted in April, 2019. The following sources were searched using English search terms for relevant grey literature documents, conference abstracts and other data sources including other websites referred to, published from 2000 onwards:

- WHO: http://www.who.int/csr/disease/meningococcal/en/; http://www.who.int/emergencies/diseases/meningitis/en/
- Centre for Disease Prevention and Control (CDC): https://www.cdc.gov/meningococcal/index.html
- Global Invasive Bacterial Vaccine-Preventable Diseases Laboratory Network: http://www.who.int/immunization/monitoring_surveillance/burden/laboratory/IBVPD/en/
- WHO Regional Office for the Eastern Mediterranean: www.emro.who.int
- Middle East Health Statistics: https://libguides.gwumc.edu/c.php?g=27767&p=170228
- Google Scholar search on meningococcal disease in the overarching regions (e.g. Middle East, Eastern Mediterranean, Eurasia and North Africa)
- The background presents a global overview of meningococcal disease, using a narrative approach. For this minor narrative review, a grey literature search was performed in June 2019. Focus in the background section was on the aetiology and transmission of disease, risk factors of meningococcal disease, and the symptoms and semiology. The websites of WHO, CDC, Google and PubMed (MEDLINE) were used to find relevant information.

**Selection procedure**

***Three-step procedure***

From the articles retrieved from PubMed (MEDLINE) and Embase.com, the relevant references were selected by a three-step selection procedure, based on:

1. Screening of title and abstract (first selection step): this step yields the articles that were assessed in full text. The major topics of the articles were assessed on relevancy for the objectives by the title and abstract. In this step, articles that seemed to contain relevant data for the objectives were selected for full-text screening, while articles that did not seem to contain relevant data were not selected for full-text assessment.
2. Screening of full article (second selection step): the articles selected during the first phase were assessed in full text. PDF-files of the original articles were downloaded and stored. Articles were included if the reported information is relevant (based on the inclusion and exclusion criteria, see Table 3) and of sufficient quality (see section 2.4).
3. Screening during data-extraction phase (third selection step): further scrutiny of the article during the data-extraction phase might have led to exclusion. For example, when articles make use of the same dataset and present identical outcome measures, the most recent or the most complete article was included.

***Inclusion and exclusion criteria***

The list of inclusion and exclusion criteria is presented below:

|  | Inclusion | - Exclusion |
| --- | --- | --- |
| Period of publication | - 2000 onwards | - Period NR |
| Study design/ type | - Prospective observational studies  (e.g. cohort studies) - Retrospective observational studies (e.g. case-control studies) - Cross-sectional studies - Economic evaluation studies - HTAs | - Meta-analysis or systematic review* - Narrative reviews - Randomised controlled trials (RCTs) - Non-pertinent publication types (e.g. abstract only, expert opinions, letters to the editor, editorials, comments) - Animal studies - Vaccine immunogenicity and safety studies - Carriage study without data on vaccination status |
| Study quality | - Sufficient methodological quality | - Insufficient methodological quality or no clear description of methodology |
| Study population | - Adults - Children - Otherwise healthy population - Immunocompromised | - (part of the) population participated in RCT |
| Study comparison | - Comparison appropriate for a specific outcome (see Table 1) |  |
| Country of study | - Countries of the AMEE region | - All other countries |
| Study outcomes/ focus of study | - Quantitative outcomes and qualitative outcomes as specified in Table 2 |  |

*Relevant meta-analyses and systematic reviews were selected during the screening of title and abstract phase. During the full-text screening phase, reference lists of good quality meta-analyses and systematic reviews were checked for possibly missed relevant individual articles. No data extraction was performed for meta-analyses or systematic reviews, only for relevant individual articles.

***Critical appraisal***

As most studies were not of a classical design suited to appraisal using existing checklists, no checklists were used to assess the quality of the articles or to calculate a total quality score. Nevertheless, some articles were excluded because of quality issues or major limitations in their design or reporting of meningococcal burden (e.g. no or unclear method section).

**Data extraction**

***Data extraction peer-reviewed literature***

Relevant data from the included articles found in the peer-reviewed were summarised using a standard data-extraction spreadsheet in Excel. Relevant information from articles that reported on the cost of meningococcal disease, cost-effectiveness of meningococcal ACWY vaccination and HTA reports was directly extracted in the corresponding section in the report and not extracted in Excel.

- One general worksheet included the study and patient characteristics, and a separate worksheet was also prepared with relevant outcomes for each objective extracted.

***Clarification Excel data extraction sheets***

- Percentages were recalculated (using Excel) to obtain one decimal place (if possible), to correct miscalculation or to (re)calculate data for subgroups.
- Three age group categories were created (children, adults, both children and adults) based on the ages provided in the articles. When articles used smaller age groups (e.g. 0 to 1 years, 2 to 5 years, 6 to 10 years and 11 to 17 years), all age groups were separately categorised as children leading to multiple rows labelled as ‘children’ in the Excel file. For outcomes with multiple rows for an age group category, a range is used to present the data per age group in the summary tables included in this report. Age groups definitions:
  - Children: all cases aged younger than 18 years. Includes age groups where the majority of the age range is within the <18 years group (e.g. 10-19 years-old).
  - Adults: all cases aged 18 years or older. Includes age groups where the majority of the age range is ≥18 years (e.g. 17-25 years old).
  - Children and adults: this label is used when nothing is reported in a study on age, when a study included cases of all ages, or when the overlap in the ‘children’ and ‘adults’ categories is large and the age category is not clear (e.g. 15-20 years or 13-100 years).

Relevant information from the grey literature was directly extracted in the corresponding section in the report and not extracted in Excel. The same applied for the figures that were extracted from the included references.

***Quality control***

The following quality control measures were put in place:

- The first 30% of titles and abstracts from the peer-reviewed literature were screened in duplicate by two independent researchers. The results were compared and discussed before the remaining references are assessed by one researcher. There was no more than 5% difference of the articles screened in duplicate between the two researchers.
- The first 10% of the full-text articles from the peer-reviewed literature were critically appraised in duplicate by two independent researchers. The results were compared and discussed early in the process. Any disagreements were adjudicated by a third researcher if necessary. There was no more than 5% difference of the articles screened in duplicate between the two researchers.
- Data extraction: the data extraction tables were compiled by researchers and reviewed by another researcher of the project.

## Table S1. Total number of cases and associated CFR, by study

| **Country** | **Population type** | **Total number of cases** | **CFR** |
| --- | --- | --- | --- |
| Afghanistan, Iran, Iraq, Egypt, Libya, Morocco, Pakistan, Sudan, Syria, and Yemen [1] | General population | 458 | 5.7–6.7 |
| Iran [2] | General population | 68 | 10.3 |
| Iran [3] | General population | 16 | 18.8 |
| Iran [4] | General population | 1370 | 0.0–100.0 |
| Iran [5] | General population | 95 | 18.9 |
| Kuwait [6] | General population | 30 | 0.0 |
| Kuwait [7] | General population | 293 | 11.3–17.5 |
| Kuwait [8] | General population | 10 | 10.0 |
| Qatar [9] | General population | 25 | 4.0 |
| Qatar [10] | General population | 2 | 0.0 |
| Pakistan [11] | General population | 30 | 10.0 |
| Pakistan [12] | General population | 105 | 9.5 |
| Saudi Arabia [13] | General population | 37 | 0.0 |
| Saudi Arabia [14] | General population | 1103 | 6.3–32.6 |
| Turkey [15] | General population | 2 | 0.0 |
| Turkey [16] | General population | 65 | 18.5 |
| Turkey [17] | General population | 85 | 3.5 |
| Turkey [18] | General population | 333 | 3.3 |
| Turkey [19] | General population | 7 | 14.3 |
| Turkey [20] | General population | 16 | 43.8 |
| Turkey [21] | General population | 5 | 0.0 |
| Turkey [22] | General population | 15 | 0.0 |
| Turkey [23] | General population | 66 | 0.0 |
| Turkey [24] | General population | 89 | 3.4 |
| Yemen [25] | General population | 20 | 20.0 |
| Yemen [26] | General population | 81 | 0.0–6.3 |
| Iran [4] | Military | 66 | 0.0–50.0 |
| Saudi Arabia [27] | Pilgrims | 105 | 0.0–50.0 |
| Saudi Arabia [28] | Pilgrims | 253 | 27.7 |
| Egypt [29] | General population | 203 | 14.3 |
| Egypt [30] | General population | 30 | 23.0 |
| Egypt [31] | General population | 314 | 13.4 |
| Morocco [32] | General population | 29 | 31.0 |
| Tunisia [33] | General population | 79 | 17.7 |

## Figure S1. Countries with/without data for each objective


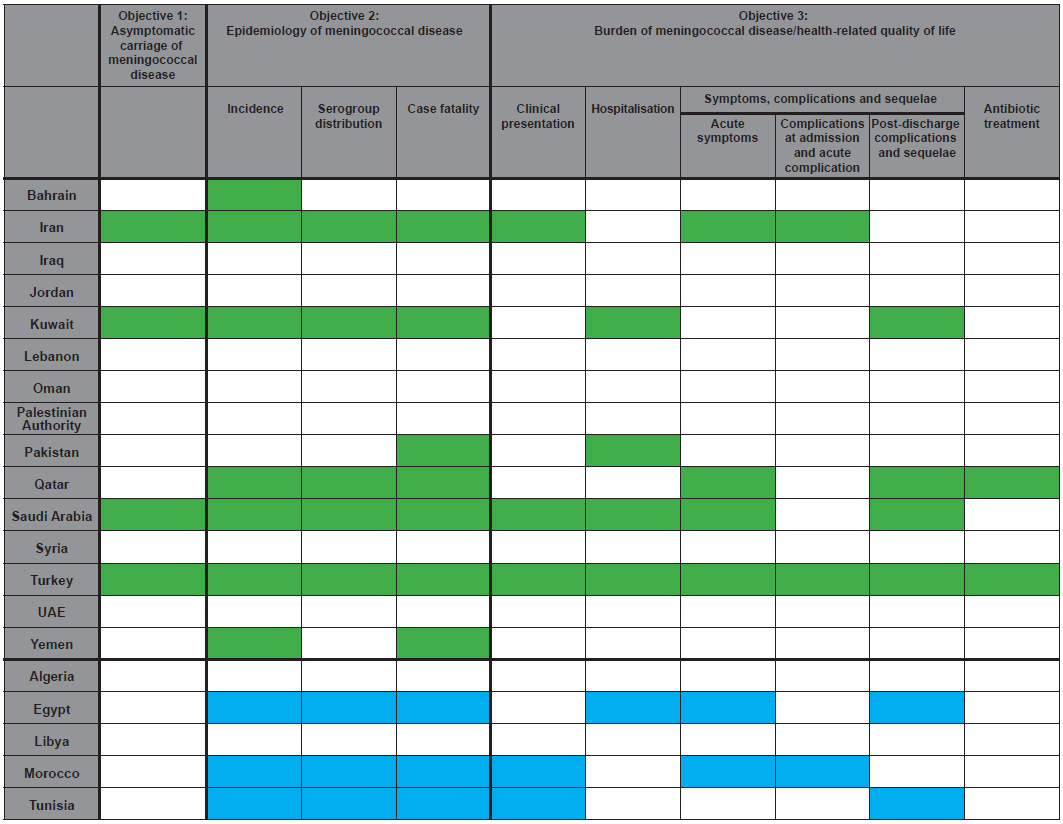


Green = data identified for countries in EM region; Blue = data identified for countries in NA region.

C&S, complications and sequelae; EM, Eastern Mediterranean; NA, North Africa; UAE, United Arab Emirates.

## References

1. Teleb N, Pilishvili T, Van Beneden C, Ghoneim A, Amjad K, Mostafa A, Estighamati AR, Smeo MN, Barkia A, ElKhatib M: **Bacterial meningitis surveillance in the Eastern Mediterranean region, 2005-2010: successes and challenges of a regional network**. *The Journal of pediatrics* 2013, **163**(1):S25-S31.

2. Mamishi S, Mostashfi Habibabadi S, Elahi B: **Clinical and laboratory manifestations of meningococcemia in children**. *Iranian journal of public health* 2006, **35**(4):49-53.

3. Attarpour-Yazdi MM, Ghamarian A, Mousaviehzadeh M, Davoudi N: **Identification of the serotypes of bacterial meningitis agents; implication for vaccine usage**. *Iranian journal of microbiology* 2014, **6**(4):211-218.

4. Tavana AM, Ataee RA: **Meningococcal meningitis control in Iran: Five year comparative study 2000-2004**. *Journal of Medical Sciences* 2009, **9**(1):51-54.

5. Berangi Z, Karami M, Mohammadi Y, Nazarzadeh M, Zahraei SM, Javidrad H, Heidari S: **Epidemiological profile of meningitis in Iran before pentavalent vaccine introduction**. *BMC Pediatr* 2019, **19**(1):370.

6. Husain EH, Al-Shawaf F, Bahbahani E, El-Nabi MH, Al-Fotooh KA, Shafiq MH, Al-Ateeqi N, Talib MAA: **Epidemiology of childhood meningitis in Kuwait**. *Medical Science Monitor* 2007, **13**(5):CR220-CR223.

7. Husain EH, Barakat M, Al-Saleh M: **Trends and variations in the epidemiology of meningococcal disease in Kuwait 1987-2013**. *Journal of infection and public health* 2015, **8**(5):441-447.

8. Sadeq H, Husain EH, Alkoot A, Atyani S, Al-fraij A, Al-Daithan A, AlSaleem T, Taher A, Alenezi M: **Childhood meningitis in Kuwait in the era of post pneumococcal conjugate vaccination: A multicenter study**. *Journal of infection and public health* 2017, **10**(6):766-769.

9. Al Maslamani E, Al Soub H, Al Maslamani M, Abu Khatab M: **Some of the meningococcal meningitis in Qatar: Epidemiology, clinical and laboratory features**. *Qatar Medical Journal* 2010, **19**(2):17-21.

10. Elsaid MF, Alsoub H, Bessisso MS, Janahi M, Elshafie S, Amina A: **Clinical presentation of acute bacterial meningitis in Qatar**. *Neurosciences* 2002, **7**(4):266-271.

11. Rabbani MA, Khan AA, Ali SS, Ahmad B, Baig SM, Khan MA, Wasay M: **Spectrum of Complications and Mortality of Bacterial Meningitis: An Experience from a Developing Country**. *Journal of the Pakistan Medical Association* 2003, **53**(12):580-583.

12. Rizvi SFS, Arif U, Khan MUR: **Frequency of etiological agents and the clinical outcome of acute bacterial meningitis**. *Pakistan Journal of Medical and Health Sciences* 2016, **10**(4):1264-1267.

13. Al-Mazrou YY, Musa EK, Abdalla MN, Al-Jeffri MH, Al-Hajjar SH, Mohamed OM: **Disease burden and case management of bacterial meningitis among children under 5 years of age in Saudi Arabia**. *Neurosciences* 2004, **9**(1):38-45.

14. Memish Z, Al Hakeem R, Al Neel O, Danis K, Jasir A, Eibach D: **Laboratory-confirmed invasive meningococcal disease: Effect of the Hajj vaccination policy, Saudi Arabia, 1995 to 2011**. *Eurosurveillance* 2013, **18**(37).

15. Gürgey A, Aytac S, Kanra G, Secmeer G, Ceyhan M, Altay C: **Outcome in children with purpura fulminans: Report on 16 patients**. *American Journal of Hematology* 2005, **80**(1):20-25.

16. Akyildiz B, Uzel N, Çitak A, Soysal D, Karaböcüoǧlu M, Üçsel R: **The factors affecting mortality in meningococcal disease**. *Cocuk Sagligi ve Hastaliklari Dergisi* 2008, **51**(1):26-30.

17. Ceyhan M, Ozsurekci Y, Gurler N, Karadag Oncel E, Camcioglu Y, Salman N, Celik M, Emiroglu MK, Akin F, Tezer H *et al*: **Bacterial agents causing meningitis during 2013-2014 in Turkey: A multi-center hospital-based prospective surveillance study**. *Human vaccines & immunotherapeutics* 2016, **12**(11):2940-2945.

18. Ceyhan M, Gürler NG, Ozsurekci Y, Keser M, Aycan AE, Gurbuz V, Salman N, Camcioglu Y, Dinleyici EC, Ozkan S *et al*: **Meningitis caused by neisseria meningitidis, hemophilus influenzae type b and streptococcus pneumoniae during 2005-2012 in Turkey: A multicenter prospective surveillance study**. *Human Vaccines and Immunotherapeutics* 2014, **10**(9):2706-2712.

19. Kepenekli E, Tuygun N, Tanir G: **Çocuklarda Invazif Meningokokkal Hastalik: 7 Hastada Klinik Özellikler ve Tedavi Yaklasiminin Degerlendirilmesi/Invasive Meningococcal Disease in Children; Assessment of Clinical Features and Treatment Options in 7 Patients**. *Cocuk Enfeksiyon Dergisi* 2008, **2**(4):152.

20. Kulcu NU, Say A, Guven F, Degirmenci S, Ozkan A, Masatli R: **Evaluation of children with invasive meningococcal disease hospitalized between 2003-2007**. *JOURNAL OF PEDIATRIC INFECTION* 2008, **2**(1):7-11.

21. Özdemir H, Tapisiz A, Çiftçi E, Ince E, Doǧru U: **Acute bacterial meningitis in children**. *Cocuk Enfeksiyon Dergisi* 2010, **4**(1):9-14.

22. Kara Uzun A, Yöney A, Kunak B, Kanik Yüksek S, Tezer H: **Evaluation of children with acute bacterial meningitis treated in two separate time periods in Turkey**. *Turkish Journal of Pediatric Disease* 2018, **12**(3):193-199.

23. Bor M, Cokugras H: **Factors associated with early complications in inpatients who were treated in our clinic between 1992 and 2011 with a diagnosis of acute bacterial meningitis**. *Turk Pediatri Ars* 2020, **55**(2):149-156.

24. Ceyhan M, Ozsurekci Y, Tanir Basaranoglu S, Gurler N, Sali E, Keser Emiroglu M, Oz FN, Belet N, Duman M, Ulusoy E *et al*: **Multicenter Hospital-Based Prospective Surveillance Study of Bacterial Agents Causing Meningitis and Seroprevalence of Different Serogroups of Neisseria meningitidis, Haemophilus influenzae Type b, and Streptococcus pneumoniae during 2015 to 2018 in Turkey**. *mSphere* 2020, **5**(2).

25. Abdulrab A, Algobaty F, Salem AK, Mohammed YAK: **Acute bacterial meningitis in adults: A hospital based study in Yemen**. *Japanese journal of infectious diseases* 2010, **63**(2):128-131.

26. Al Khorasani A, Banajeh S: **Bacterial profile and clinical outcome of childhood meningitis in rural Yemen: A 2-year hospital-based study**. *Journal of Infection* 2006, **53**(4):228-234.

27. Karima TM, Bukhari SZ, Fatani MI, Yasin KA, Al-Afif KA, Hafiz FH: **Clinical and microbiological spectrum of meningococcal disease in adults during Hajj 2000: an implication of quadrivalent vaccination policy**. *JPMA The Journal of the Pakistan Medical Association* 2003, **53**(1):3-7.

28. Lingappa JR, Al-Rabeah AM, Hajjeh R, Mustafa T, Fatani A, Al-Bassam T, Badukhan A, Turkistani A, Al-Hamdan N, Al-Jeffri M *et al*: **Serogroup W-135 meningococcal disease during the Hajj, 2000**. *Emerging infectious diseases* 2003, **9**(6):665-671.

29. Afifi S, Wasfy MO, Azab MA, Youssef FG, Pimentel G, Graham TW, Mansour H, Elsayed N, Earhart K, Hajjeh R *et al*: **Laboratory-based surveillance of patients with bacterial meningitis in Egypt (1998-2004)**. *European Journal of Clinical Microbiology and Infectious Diseases* 2007, **26**(5):331-340.

30. Youssef FG, El-Sakka H, Azab A, Eloun S, Chapman GD, Ismail T, Mansour H, Hallaj Z, Mahoney F: **Etiology, antimicrobial susceptibility profiles, and mortality associated with bacterial meningitis among children in Egypt**. *Annals of epidemiology* 2004, **14**(1):44-48.

31. Mobarak EI: **Trend, features and outcome of meningitis in the Communicable Diseases hospital, Alexandria, Egypt, 1997-2006**. *The Journal of the Egyptian Public Health Association* 2012, **87**(1-2):16-23.

32. Loutfi A, M ELH, Jayche S, Mohammed L, Asmaa A, Lhou A, Dahou B, Omar Touhami Ahami A: **Epidemiological, Cytochemical and Bacteriological Profile of Meningitis among Adults and Children in North West of Morocco**. *Pakistan journal of biological sciences : PJBS* 2020, **23**(7):891-897.

33. Smaoui H, Saguer A, Bouziri A, Fourati S, Chahed MK, Ben Jaballah N, Ben Bousnina S, Barsaoui S, Sammoud A, Ben Becheur S *et al*: **[Neisseria meningitidis invasive infections in children in Tunis: about 79 cases]**. *Archives de l'Institut Pasteur de Tunis* 2011, **88**(1-4):35-41.
